# Supplementary material for: Clinical Impact of Systematic Assessment and Psychoeducation in Specialized Treatment of Adolescents with Severe Functional Somatic Disorders: Results from the AHEAD Study
Source: Children (Basel). 2023 Jun 22;10(7):1101. doi: 10.3390/children10071101 (PMC10378561; doi:10.3390/children10071101)
Supplement: Supplementary file 1 [file children-10-01101-s001.zip › children-2432436-supplementary.pdf]

Supplementary Materials

Figure S1. Chronological overview of health care contacts and social events

**Symptoms Examinations Diagnoses Treatment**	Year	**Social events**
Chronological listing (starting at birth) of all information regarding symptoms, health care contacts, diagnoses and interventions.<br><br>Treatment includes pharmacological and non-pharmacological (e.g. physiotherapy, psychotherapy) but also alternative treatment.	↓	Listing of important social events, both positive and challenging (e.g. schooling, social and leisure activities, parental divorce, bullying, loss of loved ones).
Example:		*Example:*
2012 Start of abdominal pain, several contacts to GP with no clear explanation		*2012 Emerging problems in school, difficulties reading*
2014 Diagnosed with dyslexia		
2018 Emergency room: Sudden onset of severe headache. Clinical examination and MRI normal		*2018 Loss of aunt. Family in crisis*
2019 Pediatric department: Headache continues, and new symptoms emerge including extreme fatigue and pain in several joints. X-rays, blood and urin tests normal.		*2021 Start of high-school*
2023 Second opinion at private hospital		*2023 Difficulties keeping up in high-school. High abscence*

GP: general practitioner; MRI: Magnetic Resonance Imaging

Figure S2 Experience of assessment divided by randomization group

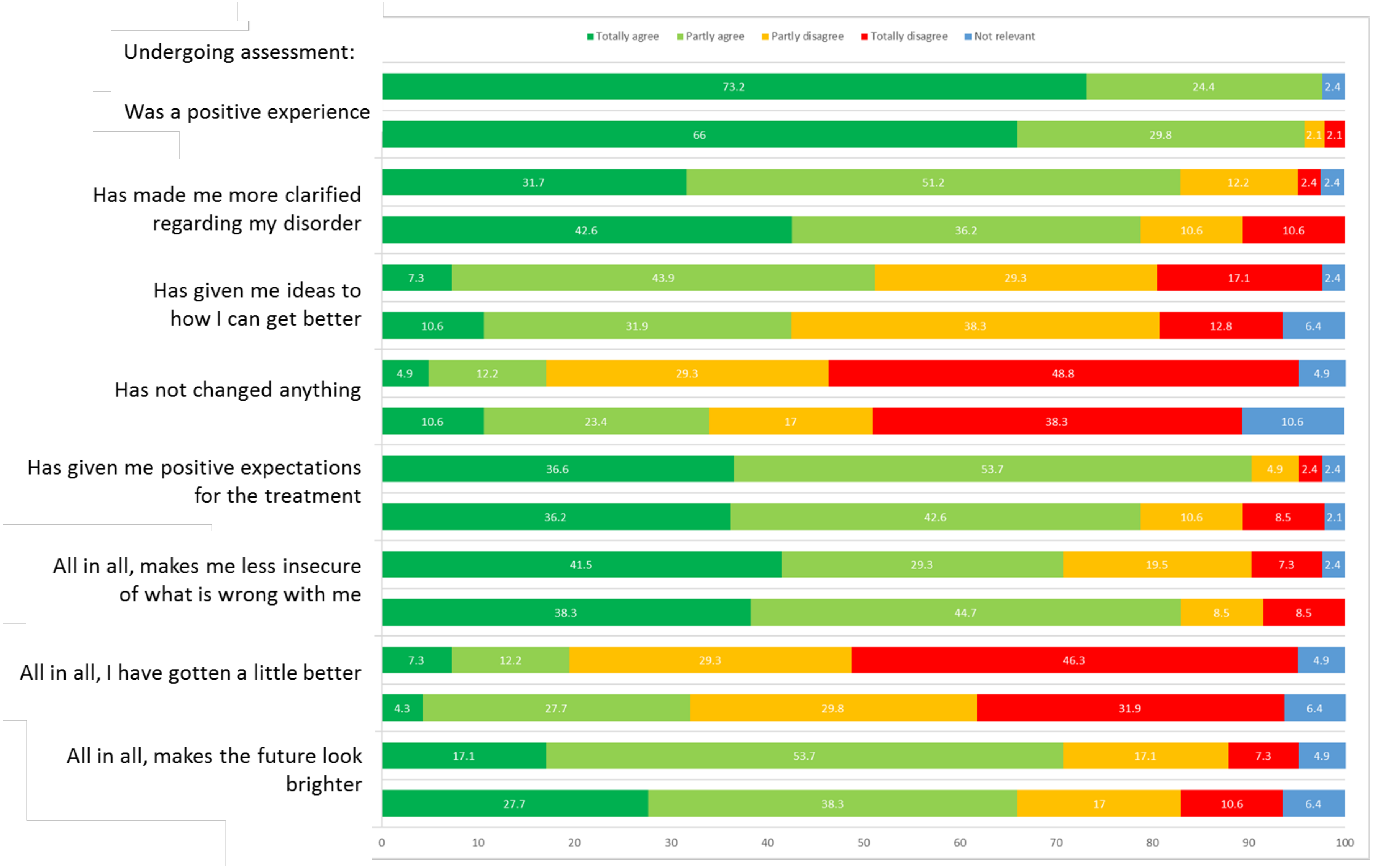

Numbers in figure represent percentage
AHEAD n=41 (missing n=3), EUC n=47 (missing n=0)

Table S1 Mean and SD at T0 and T1

		AHEAD (T0)		AHEAD (T1)		EUC (T0)		EUC (T1)	
		Mean	SD	Mean	SD	Mean	SD	Mean	SD
**Primary outcome**									
Physical health	SF-36 (15-65)	36.58	5.83	36.43	6.71	37.10	7.79	38.48	6.82
**Secondary outcomes**									
Symptom severity	SCL-som (0-4)	2.01	0.74	1.81	0.63	1.81	0.81	1.67	0.69
Illness worry	Whiteley-6-R (0-4)	1.62	1.15	1.03	0.97	1.71	1.06	1.00	0.89
Mental health	SF-36 MCS	35.66	16.49	37.57	15.17	35.80	11.75	36.57	10.64
**Treatment targets**									
Illness perception	B-IPQ (0-80)	52.27	7.68	49.02	10.11	51.37	8.07	45.98	9.33
Illness-related behaviour	BRIQ- All or nothing (6-30)	19.41	4.47	18.37	5.19	18.85	4.78	18.36	3.70
	BRIQ- Limiting (7-35)	23.50	5.19	22.26	4.93	23.64	5.48	22.04	5.17
Psychological inflexibility	AFQ-Y8 (0-32)	11.57	9.20	11.38	8.46	13.34	7.36	11.97	7.01
	PIPS- Avoidance (8-56)	35.16	9.97	31.48	10.13	34.53	10.98	32.30	10.55
	PIPS- Fusion (4-28)	21.93	3.88	21.33	4.84	22.06	4.96	20.77	5.01

T0: Questionnaires at baseline prior to assessment
T1: Questionnaires at approximately 2 months prior to specialized treatment
